# Necro-inflammatory activity grading in chronic viral hepatitis with three-dimensional multifrequency MR elastography

**DOI:** 10.1038/s41598-021-98726-x

**Published:** 2021-09-29

**Authors:** Philippe Garteiser, Gwenaël Pagé, Gaspard d’Assignies, Helena S. Leitao, Valérie Vilgrain, Ralph Sinkus, Bernard E. Van Beers

**Affiliations:** 1 Laboratory of Imaging Biomarkers, Center for Research on Inflammation, UMR 1149 Inserm, Université de Paris, 75018 Paris, France; 2 Department of Radiology, Beaujon University Hospital Paris Nord, AP-HP, 92110 Clichy, France; 3 Laboratory for Vascular Translational Science, UMR 1148 Inserm, Université de Paris, 75018 Paris, France

**Keywords:** Magnetic resonance imaging, Hepatology, Diagnostic markers, Imaging techniques

## Abstract

The purpose of this study was to assess the diagnostic value of multifrequency MR elastography for grading necro-inflammation in the liver. Fifty participants with chronic hepatitis B or C were recruited for this institutional review board-approved study. Their liver was examined with multifrequency MR elastography. The storage, shear and loss moduli, and the damping ratio were measured at 56 Hz. The multifrequency wave dispersion coefficient of the shear modulus was calculated. The measurements were compared to reference markers of necro-inflammation and fibrosis with Spearman correlations and multiple regression analysis. Diagnostic accuracy was assessed. At multiple regression analysis, necro-inflammation was the only determinant of the multifrequency dispersion coefficient, whereas fibrosis was the only determinant of the storage, loss and shear moduli. The multifrequency dispersion coefficient had the largest AUC for necro-inflammatory activity A ≥ 2 [0.84 (0.71–0.93) vs. storage modulus AUC: 0.65 (0.50–0.79), *p* = 0.03], whereas the storage modulus had the largest AUC for fibrosis F ≥ 2 [AUC (95% confidence intervals) 0.91 (0.79–0.98)] and cirrhosis F4 [0.97 (0.88–1.00)]. The measurement of the multifrequency dispersion coefficient at three-dimensional MR elastography has the potential to grade liver necro-inflammation in patients with chronic vial hepatitis.

## Introduction

Ultrasound and magnetic resonance (MR) elastography are increasingly used for staging liver fibrosis in patients with hepatitis B and C^1–4^. Most patients with chronic viral hepatitis and liver fibrosis also have necro-inflammatory activity. Necro-inflammation has potential prognostic and therapeutic consequences^5–8^.

Although MR elastography accurately stages liver fibrosis, its role in grading liver necro-inflammation remains debated. Necro-inflammation may increase liver stiffness in diffuse liver diseases^4,9,10^, but its influence on liver stiffness is less prominent than that of fibrosis^4,11,12^.

With three-dimensional MR elastography the complete wave field is measured to calculate the viscoelastic properties of the liver^2,13^. These parameters include first, the magnitude of the complex shear modulus |G*|, also called shear stiffness; second, the storage G′ and loss G” moduli (real and imaginary parts of the complex-valued shear modulus) which reflect elasticity and viscosity respectively; and third the damping ratio ζ = G″/(2·G′) related to the viscosity to elasticity ratio^14,15^.

Moreover, with the advent of rapid acquisition schemes for multifrequency MR elastography, the exploration of the frequency behavior of liver disease has become clinically feasible^3,16,17^. The biomechanical moduli of a tissue have a frequency dependence which is best described by a power law^11,18,19^. The exponent of the power law or multifrequency dispersion coefficient is an indicator of tissue architecture and is influenced by the solid/liquid composition of tissue^18,20,21^.

These additional viscoelastic parameters at MR elastography may help assessing tissue activity. With monofrequency data acquisition, it was shown that the damping ratio and loss modulus are useful to differentiate between simple steatosis and steatohepatitis in mice and patients^15,22,23^. Using multifrequency data acquisition, it was observed that the dispersion coefficient decreased in the brain of patients with chronic neuro-inflammation and in obese rat pancreas containing fibro-inflammatory complexes^24,25^.

The value of multifrequency MR elastography for diagnosing hepatic necro-inflammation and distinguishing it from fibrosis has not yet been assessed to the best of our knowledge. The aim of our study was to assess the diagnostic value of three-dimensional multifrequency MR elastography for necro-inflammatory activity grading in patients with chronic viral hepatitis.

## Results

### Clinical and histopathologic parameters

**Figure 1:**
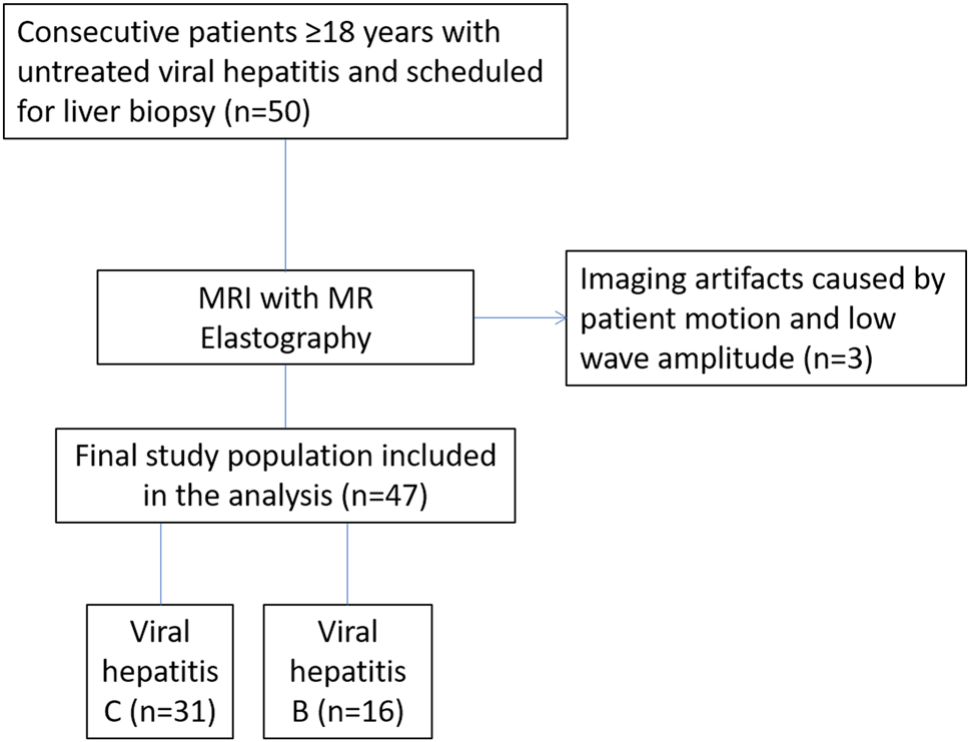
study flowchart shows patients with inclusion and exclusion criteria.

Patient age ranged from 25 to 68 years [median: 47 years, 95% confidence interval: (40–52 years)] and 73% of the patients were men. Eighteen patients had chronic viral hepatitis B and 32, chronic viral hepatitis C. The data of three patients (hepatitis B: n = 2, hepatitis C: n = 1) were excluded because of patient motion during the MR elastography examination and low wave amplitude or low wave amplitude (Fig. 1). At histopathology, the necro-inflammatory grade was A0 in 7 patients (15%), A1 in 27 patients (57.5%), A2 in 10 patients (21%) and A3 in 3 patients (6.5%). Five patients (11%) had F0 fibrosis, 19 patients (40%) F1, 9 patients (19%) F2, 9 patients (19%) F3, and 5 patients (11%) F4. Thirteen patients (28%) had substantial inflammation ≥ A2 and 23 patients (49%) had substantial fibrosis ≥ F2 (Supplementary table 1).

### Reproducibility

Shear stiffness measurements displayed intraclass correlation coefficient of 0.994 [95% confidence interval (0.989–0.997)], interobserver reproducibility coefficient of 9.2% and bias of − 1.4%. The other biomechanical parameters displayed similar levels of reproducibility (Table 1).

**Table 1:** Interobserver reproducibility of the biomechanical parameters.

Parameter	Intraclass correlation coefficient [95% confidence interval]	Bland–Altman bias (%)	Reproducibility coefficient (%)
Shear modulus, G*	0.994 [0.989–0.997]	− 1.4	9.2
Storage modulus, G′	0.995 [0.990–0.997]	− 1.3	7.9
Loss modulus, G″	0.985 [0.972–0.992]	− 1.3	15.3
Damping ratio, ζ	0.954 [0.918–0.974]	0.0	12.4
Multifrequency dispersion coefficient, γ	0.950 [0.721–0.983]	− 5.2	16.4

### Biomechanical parameters versus activity and fibrosis

**Figure 2:**
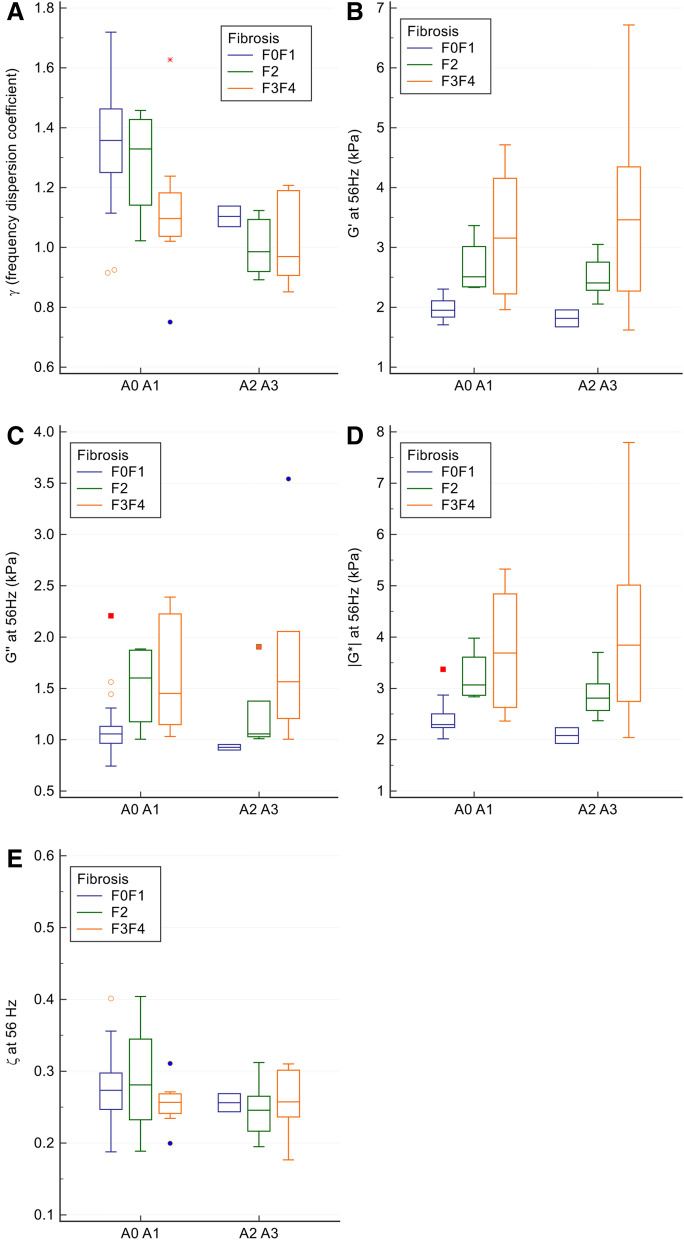
Boxplots (boxes: 1st to 3rd quartiles, whiskers: 1st quartile—1.5 × interquartile range to 3rd quartile + 1.5 × interquartile range, horizontal line: median) of multifrequency dispersion coefficient (γ, panel **A**), storage modulus (G′, panel **B**), loss modulus (G″, panel **C**), shear modulus (|G*|, panel **D**) and damping ratio (ζ, panel **E**) at 56 Hz in patients with increasing necro-inflammation score. For the two necro-inflammatory activity groups, boxplots are subdivided relative to fibrosis scores. Only multifrequency dispersion coefficient differs significantly between patients without and with substantial activity (*p* = 0.0003).

**Figure 3:**
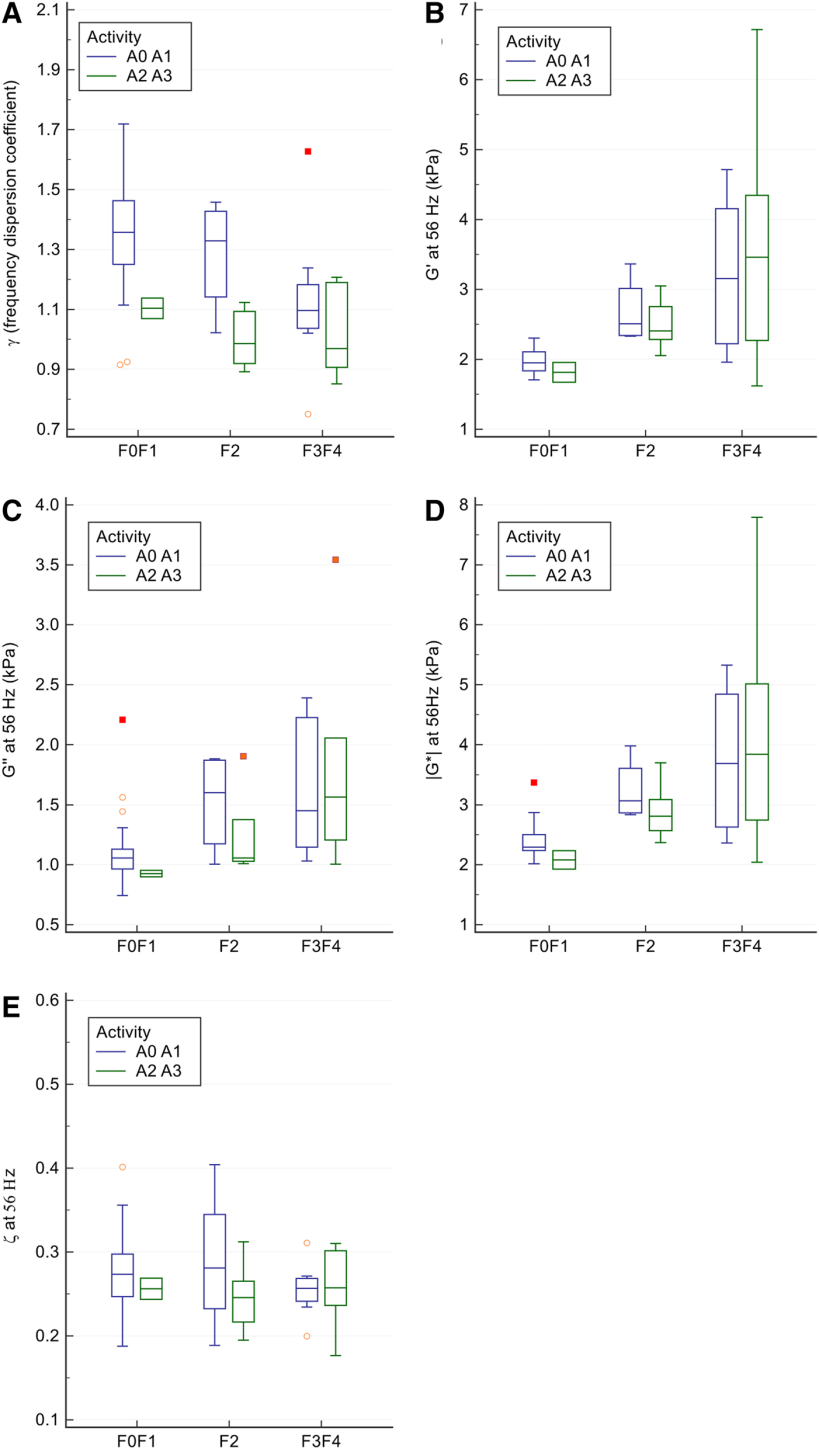
Boxplots (boxes: 1st to 3rd quartiles, whiskers: 1st quartile—1.5 × interquartile range to 3rd quartile + 1.5 × interquartile range, horizontal line: median) of multifrequency dispersion coefficient (γ, panel **A**) and monofrequency (56 Hz) storage modulus (G′, panel **B**), loss modulus (G″, panel **C**), shear modulus (|G*|, panel **D**) and damping ratio (ζ, panel **E**) in patients with increasing fibrosis. For each fibrosis group, boxplots are subdivided relative to the necro-inflammatory activity. Frequency dispersion coefficient decreases significantly (*p* = 0.003) with increasing fibrosis score, whereas storage, loss and shear moduli increase significantly with fibrosis score (*p* = 0.000008, *p* = 0.001, and *p* = 0.00002, respectively). Damping ratio does not differ significantly between groups.

**Figure 4:**
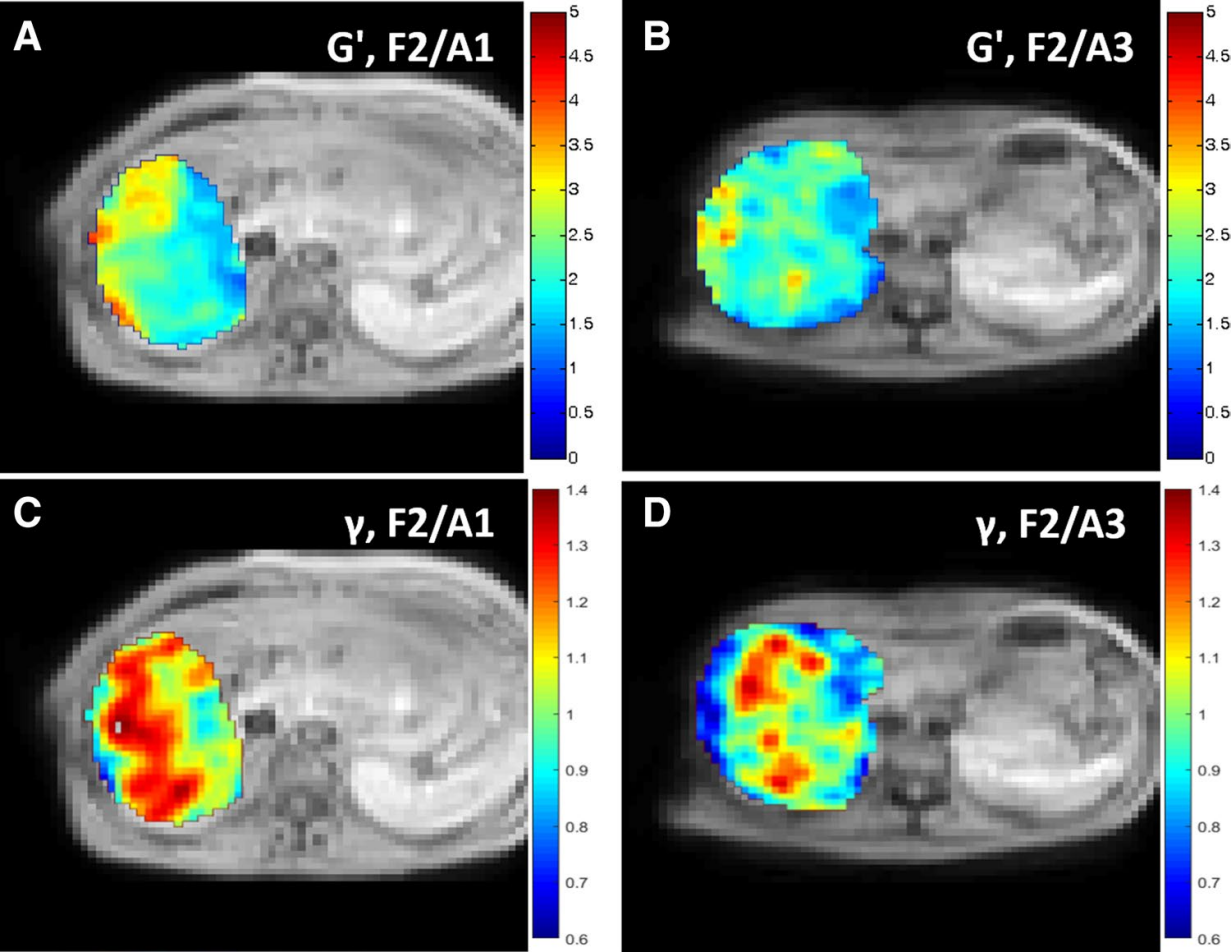
Parametric maps of storage modulus G′ (**A**,**B**) and multifrequency dispersion coefficient γ (**C**,**D**) in patient with F2/A1 histological score (**A**,**C**) and patient with F2/A3 score (**B**,**D**). Storage modulus is similar (mean storage modulus: 2.4 kPa in (**A**) versus mean 2.3 kPa in (**B**) in the two patients with same F2 score, whereas multifrequency dispersion coefficient is lower in the second patient with A3 activity than in the first patient with A1 activity (mean multifrequency dispersion coefficient: 1.46 in (**D**) versus 0.99 in (**C**).

The biomechanical parameter graphs according to the histological classification of activity and fibrosis are shown in Figs. 2 and 3 (and in detail in supplementary figs. S1 and S2) and clinical examples are illustrated in Fig. 4. Regarding necro-inflammation, only the multifrequency dispersion coefficient differed significantly between patients without and with substantial activity (A0–A1: 1.3 ± 0.2 vs. A2–A3: 1.0 ± 0.1, *p* = 0.0003). The corresponding values for the storage modulus were 2.3 ± 0.8 kPa versus 2.9 ± 1.4 kPa, *p* = 0.11, for the loss modulus 1.3 ± 0.4 kPa versus 1.5 ± 0.7 kPa, *p* = 0.70, for the shear modulus 2.8 ± 0.9 kPa versus 3.4 ± 1.6 kPa, *p* = 0.27, and for the damping ratio 0.3 ± 0.1 kPa versus 0.3 ± 0.04 kPa, *p* = 0.19 (Figs. 2, 3).

The multifrequency dispersion coefficient decreased with increasing necro-inflammation and fibrosis, whereas the monofrequency storage, loss and shear moduli increased significantly with increasing fibrosis and necro-inflammation scores The storage modulus showed the highest differences between groups of increasing fibrosis (F0–F1: 2.0 ± 0.2 kPa, F2: 2.6 ± 0.4 kPa and F3–F4: 3.4 ± 1.4 kPa, Kruskal–Wallis *p* = 0.000008), versus the loss modulus (F0–F1: 1.1 ± 0.3 kPa, F2: 1.4 ± 0.4 kPa and F3–F4: 1.7 ± 0.7 kPa, Kruskal–Wallis *p* = 0.001), the shear modulus (F0–F1: 2.4 ± 0.3 kPa, F2: 3.0 ± 0.5 kPa and F3–F4: 3.9 ± 1.6 kPa, Kruskal–Wallis *p* = 0.00002), and the multifrequency dispersion coefficient (F0–F1: 1.3 ± 0.2, F2: 1.1 ± 0.2 and F3–F4: 1.1 ± 0.2, Kruskal–Wallis *p* = 0.003). The damping ratio did not differ between the groups with increasing fibrosis (F0–F1: 0.3 ± 0.1, F2: 0.3 ± 0.1 and F3–4: 0.3 ± 0.04, Kruskal–Wallis *p* = 0.56) (Fig. 4).

### Correlations

At univariate analysis, significant correlation was observed between the multifrequency dispersion coefficient and necro-inflammatory activity (*r* = - 0.63, *p* < 0.0001). The only other viscoelastic parameter correlated to activity was the storage modulus, although with lower correlation coefficient (*r* = 0.34) and less significance (*p* = 0.02). In contrast, all viscoelastic parameters, except the damping ratio, displayed significant correlations with fibrosis (Table 2). The storage modulus showed the best correlation with fibrosis (*r* = 0.65, *p* < 0.0001).

**Table 2:** Spearman correlations (*r*) between histological features, biological data and biomechanical parameters.

Parameter	Fibrosis	Necro-inflammatory activity	ALT*	AST*
Storage modulus (G′)	0.65 (*p* < 0.0001)	0.34 (*p* = 0.203)	0.43 (*p* = 0.0025)	0.51 (*p* = 0.0003)
Loss modulus (G″)	0.49 (*p* = 0.0004)	0.10 (*p* = 0.49)	0.20 (*p* = 0.18)	0.35 (*p* = 0.0165)
Shear modulus (G*)	0.63 (*p* < 0.0001)	0.24 (*p* = 0.11)	0.31 (*p* = 0.0328)	0.44 (*p* = 0.0019)
Damping ratio (ζ)	− 0.19 (*p* = 0.21)	− 0.24 (*p* = 0.11)	− 0.17 (*p* = 0.26)	− 0.08 (*p* = 0.58)
Frequency dispersion coefficient	− 0.50 (*p* = 0.0004)	− 0.63 (*p* < 0.0001)	− 0.50 (*p* = 0.0004)	− 0.50 (*p* = 0.0003)

*ALT* alanine aminotransferase, *AST* aspartate aminotransferase.

The serum aminotransferase levels were correlated with the multifrequency dispersion coefficient (ALT: *r* = - 0.50, *p* = 0.0004; AST: *r* = - 0.50, *p* = 0.0003), and with the shear and storage moduli (ALT: *r* = 0.31, *p* = 0.03; AST: *r* = 0.44, *p* = 0.002 for shear modulus, and ALT: *r* = 0.43, *p* = 0.0025; AST: *r* = 0.51, *p* = 0.0003 for storage modulus). Moreover, there was a significant correlation (*r* = 0.54, *p* < 0.0001) between necro-inflammation and fibrosis.

At stepwise multivariate analysis, necro-inflammation was the only independent determinant of the multifrequency dispersion coefficient (r_partial_ = − 0.60, *p* < 0.0001), whereas fibrosis was a determinant of the shear, storage and loss moduli (r_partial_ = 0.66, *p* < 0.0001, r_partial_ = 0.68, *p* < 0.0001, and r_partial_ = 0.54, *p* < 0.0001, respectively). The damping ratio was not affected by any of the tested parameters (Table 3).

**Table 3:** Multivariate analysis of the potential influence of the histological features (activity, fibrosis) and biological data (alanine aminotransferase, aspartate aminotransferase) on the biomechanical parameters.

Parameter	Explaining factor	*p* value for the variable	r_partial_	F ratio (*p* value) for the model
Storage modulus (G′)	Fibrosis	< 0.0001	0.68	33.5 (*p* < 0.0001)
Loss modulus (G″)	Fibrosis	< 0.0001	0.54	15.9 (*p* = 0.0003)
Shear modulus (G*)	Fibrosis	< 0.0001	0.66	29.5 (*p* < 0.00001)
Damping ratio (ζ)	None	n.a	n.a	n.a
Frequency dispersion coefficient	Activity	< 0.001	− 0.60	21.8 (*p* < 0.0001)

*n.a*. not assessed.

### Diagnostic performance

**Figure 5:**
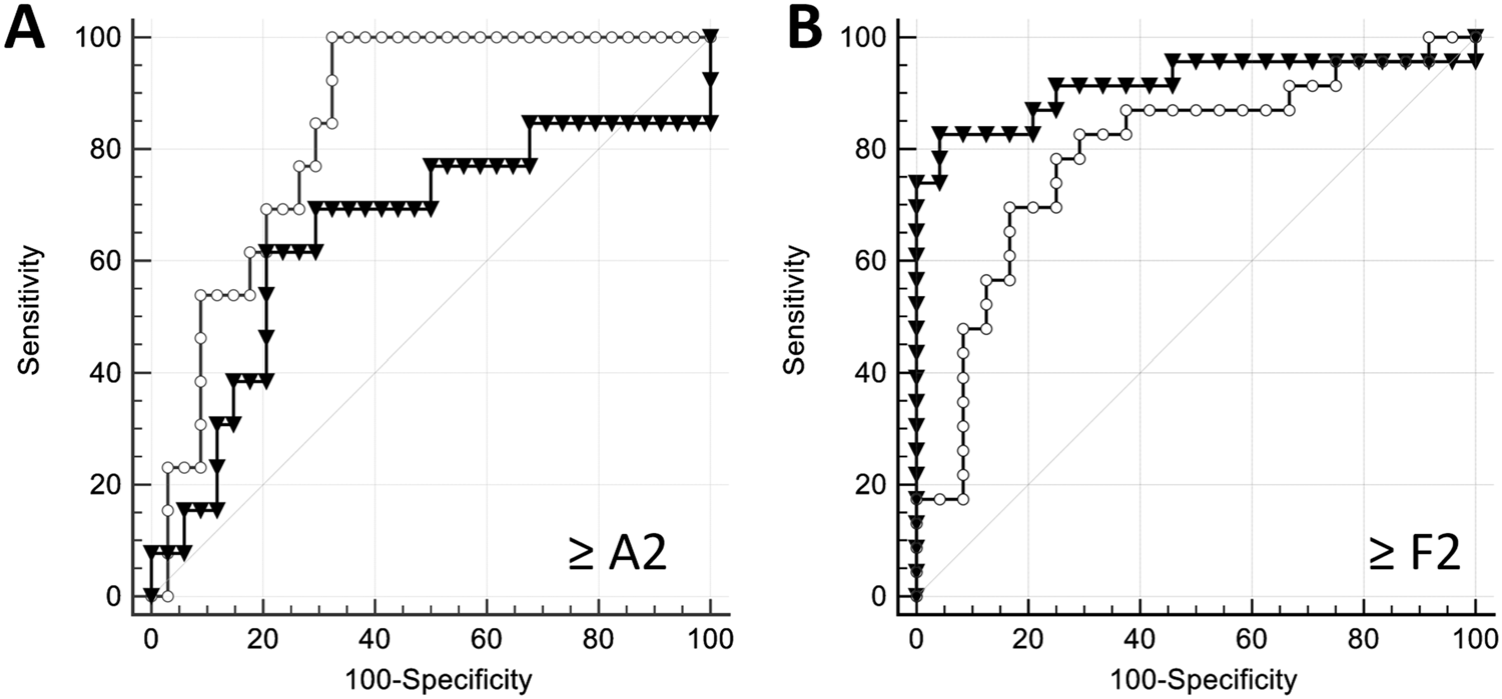
Receiver operating characteristics curves of storage modulus at 56 Hz (triangles) and multifrequency dispersion coefficient (circles) for substantial necro-inflammation ≥ A2 (**A**) and substantial fibrosis ≥ F2 (**B**). Multifrequency dispersion coefficient has significantly larger AUC than storage modulus for diagnosing necro-inflammation ≥ A2 (AUC of 0.84 vs. 0.65, *p* = 0.03), whereas storage modulus has larger AUC than multifrequency dispersion coefficient for diagnosing fibrosis ≥ F2 (AUC of 0.91 vs. 0.79, *p* = 0.18).

At ROC analysis, the multifrequency dispersion coefficient was the only parameter among the biomechanical and biological parameters displaying high AUC (≥ 0.8) for any activity grade [AUC (95% confidence interval) 0.86 (0.73–0.85), *p* < 0.0001 for A ≥ 1; 0.84 (0.71–0.93), *p* < 0.0001 for A ≥ 2; and 0.88 (0.75–0.96), *p* < 0.0001 for A = 3] (supplementary table 2. The AUC of the multifrequency dispersion coefficient for substantial activity (A ≥ 2) was significantly larger than the AUC of the storage modulus (0.84 vs. 0.65, *p* = 0.03) (Fig. 5).

At a threshold value of 1.2, the sensitivity and specificity of the multifrequency dispersion coefficient for substantial activity A ≥ 2 were 100% and 68% respectively with their positive and negative predictive values being 54% and 100% respectively. With a threshold value of 2.3 kPa, the corresponding figures for the storage modulus were 62% sensitivity, 79% specificity, 53% positive predictive value, and 84% negative predictive value (Supplementary table 2).

The storage modulus had the largest AUC for staging substantial fibrosis F ≥ 2 [0.91 (0.79–0.98), *p* < 0.0001] (Table 4). In comparison, the multifrequency dispersion coefficient had AUC of 0.79 [0.65–0.90] (*p* < 0.0001) for substantial fibrosis. The difference of AUC between the storage modulus and the multifrequency dispersion coefficient was not statistically significant (*p* = 0.18) (Fig. 5). For cirrhosis (F = 4), the storage modulus and the shear modulus had similar high AUC [0.97 (0.88–1.00), *p* < 0.0001]. In contrast, the multifrequency dispersion coefficient did not have a significant AUC [0.57 (0.41–0.71), *p* = 0.65].

**Table 4:** Areas under the receiver operating characteristic curves of the biomechanical parameters and serum aminotransferase levels in necro-inflammatory activity grading and fibrosis staging.

	A ≥ 1		A ≥ 2		A = 3	
	AUC (95% C.I.)	*p* value	AUC (95% C.I.)	*p* value	AUC (95% C.I.)	*p* value
G′	0.73 [0.58–0.85]	0.0064	0.65 [0.50–0.79]	0.13	0.83 [0.69–0.92]	< 0.0001
G″	0.56 [0.41–0.71]	0.53	0.54 [0.39–0.68]	0.71	0.73 [0.58–0.85]	0.1027
G*	0.66 [0.50–0.79]	0.07	0.60 [0.45–0.74]	0.31	0.78 [0.64–0.89]	0.0061
ζ	0.65 [0.50–0.79]	0.14	0.62 [0.47–0.76]	0.17	0.59 [0.44–0.73]	0.64
γ	0.86 [0.73–0.95]	< 0.0001	0.84 [0.71–0.93]	< 0.0001	0.88 [0.75–0.96]	< 0.0001
ALT	0.80 [0.64–0.96]	0.0002	0.75 [0.57–0.92]	0.0051	0.74 [0.67–0.80]	< 0.0001
AST	0.80 [0.73–0.85]	< 0.0001	0.76 [0.69–0.82]	< 0.0001	0.80 [0.74–0.85]	< 0.0001

	F ≥ 2		F ≥ 3		F = 4	
	AUC (95% C.I.)	*p* value	AUC (95% C.I.)	*p* value	AUC (95% C.I.)	*p* value
G′	0.91 [0.79–0.98]	< 0.0001	0.81 [0.67–0.91]	0.0001	0.97 [0.88–1.00]	< 0.0001
G″	0.79 [0.64–0.89]	< 0.0001	0.80 [0.65–0.90]	< 0.0001	0.93 [0.81–0.98]	< 0.0001
G*	0.89 [0.77–0.96]	< 0.0001	0.81 [0.67–0.91]	0.0001	0.97 [0.88–1.00]	< 0.0001
ζ	0.59 [0.44–0.73]	0.28	0.58 [0.43–0.72]	0.37	0.60 [0.44–0.74]	0.44
γ	0.79 [0.65–0.90]	< 0.0001	0.75 [0.60–0.87]	0.0013	0.57 [0.41–0.71]	0.65
ALT	0.85 [0.79–0.90]	< 0.0001	0.82 [0.75–0.87]	< 0.0001	0.78 [0.72–0.84]	< 0.0001
AST	0.86 [0.80–0.91]	< 0.0001	0.85 [0.79–0.90]	< 0.0001	0.81 [0.75–0.87]	< 0.0001

G′, storage modulus; G″, loss modulus; |G*|, shear modulus; ζ, damping ratio; γ, frequency dispersion coefficient; ALT, alanine aminotransferase; AST, aspartate aminotransferase.

At a threshold value of 1.2, the sensitivity and specificity of the multifrequency dispersion coefficient for substantial fibrosis F ≥ 2 were 83% and 71%, respectively with a positive predictive value of 73% and negative predictive value 81%. With a threshold value of 2.2 kPa, the corresponding figures for the storage modulus were as follows: 83% sensitivity, 96% specificity, 95% positive predictive value and 85% and negative predictive value (supplementary table 2).

## Discussion

In patients with chronic viral hepatitis, we have observed that the wave dispersion coefficient at multifrequency MR elastography has high diagnostic accuracy for grading necro-inflammation and we have confirmed that the monofrequency visco-elastic parameters, especially the storage and shear moduli have high diagnostic accuracy for determining fibrosis stage^12,26^. The model independent power law exponent used to assess wave dispersion in our work is a measurement that describes how the shear modulus varies with frequency^18,27^. The decrease of the multifrequency wave dispersion coefficient in hepatic necro-inflammation might be explained by an increase of edema and angiogenesis, and/or by a change of the collagen network topology^11,18,20,21^. Previous studies reporting on multifrequency dispersion in inflammation are sparse. Decreased power law dispersion at MR elastography has been reported in chronic neuro-inflammation and in the inflamed pancreas of obese rats^24,25^.

Monofrequency elasticity and stiffness measurements have been shown to be variably influenced by liver inflammatory activity^4,9^. Overall, it is accepted that early increase of stiffness and elasticity in chronic liver disease occurs before substantial matrix deposition^9^.

In chronic liver diseases, however, inflammation usually increases stiffness less than fibrosis does^4,28^. In contrast, increase in stiffness can be erroneously interpreted as elevation in fibrosis stage during acute inflammatory flares in patients with viral hepatitis^29^. Our results suggest that better discrimination between inflammation and fibrosis can be obtained with the multifrequency dispersion coefficient than with monofrequency stiffness measurements.

Viscosity related parameters, including loss modulus and damping ratio, have been reported to change at monofrequency MR elastography in NASH. The loss modulus and the damping ratio increased in animal models of NASH^15,23^, whereas the damping ratio decreased in patients with NASH relative to simple steatosis^22^. In our study of patients with chronic viral hepatitis, we did not observe these findings, and the damping ratio was not relevant for diagnosing inflammation. Similar results about insensitivity of damping ratio to inflammation have been described in a study including 40 patients with hepatitis C viral infection and 5 patients with steatohepatitis related to human immune deficiency virus infection^30^. The variable reported influence of inflammation on the damping ratio might be explained by differences in cause and duration of chronic liver disease, and by differences in MR acquisition parameters.

In contrast to what we observed in our study, it has been reported that the damping ratio and the multifrequency wave dispersion coefficient can be related, especially if it is assumed that the tissue has a specific fractal hierarchy corresponding to a springpot model^31^. In that case, the dispersion coefficient has values between 0 (pure solid) and 1 (pure liquid). Here, we observed dispersion coefficients above one, in violation of the springpot model. However, this model might not be adequate for assessing chronic liver diseases^21^. Moreover, according to the unifying theory for shear and compression waves, the dispersion coefficient of the shear modulus may exceed one under high frequency assumption^32^. This would be consistent with the results of high multifrequency dispersion coefficient observed here and elsewhere^18,27^.

Assessing disease severity, i.e. liver fibrosis and inflammation, is clinically relevant in patients with chronic viral hepatitis. This is especially true in patients with chronic hepatitis B, whose treatment is based on the level of viremia, the severity of liver fibrosis and inflammation^5,6,33^. Liver fibrosis can be reliably evaluated with elastography, but development of non-invasive methods to assess liver inflammation are still needed. In patients with chronic hepatitis B, serum transaminase levels (especially AST) are widely used for this purpose. Studies have, however, shown limited correlations between serum ALT levels and histological activity^34,35^.

In patients with chronic hepatitis C, precise assessment of inflammation is less needed because universal treatment is currently recommended. However, assessment of disease severity with elastography is still recommended before treatment and this assessment can be biased when transaminase levels are elevated^8^. These considerations underscore the potential clinical relevance of multifrequency MR elastography to assess fibrosis and inflammation in patients with chronic viral hepatitis.

Our study had several limitations. First, the study was performed with a relatively small number of patients. However, differences in diagnostic performance of necro-inflammation were observed between multi- and monofrequency parameters. The limited number of patients prevented us from analyzing the imaging markers separately in hepatitis B and C patients and necessitated the pooling of the patients without or with substantial necro-inflammation (A0/A1 vs. A2/A3) and with no/mild fibrosis (F0/F1) versus moderate fibrosis (F2) and advanced fibrosis (F3/F4). However, similar pooling has been recommended in previous large clinical studies and may have clinical relevance^33,36,37^. Indeed, substantial necro-inflammation (A2/A3) is considered to be clinically significant^33^ and advanced fibrosis (F3/F4) without cirrhotic decompensation is considered to correspond to clinically advanced compensated chronic liver disease^37^.

Second, only three mechanical frequencies were sampled. Using higher number of frequencies might be helpful in providing more exact estimates of the multifrequency dispersion coefficient. For instance, Asbach et al. used four frequencies in their study on liver fibrosis^3^, although with a lesser range (37.5 Hz, vs. 56 Hz in our study).

Third, the AUCs of the multifrequency dispersion coefficient were not statistically larger than those of the transaminase levels to assess necro-inflammatory severity. However, the multifrequency dispersion coefficient was the only coefficient with high AUC (> 0.8) for each activity grade. The lack of statistically significant difference may be related to the limited number of patients in our study. The diagnostic accuracy of the multifrequency dispersion coefficient relative to that of transaminase levels and other blood biomarkers of inflammation should be further assessed in large clinical trials.

Finally, only semi-quantitative histological scores were available for the reference examination. More advanced histological methods will be needed to explore the relationships between the biomechanical parameters and edema, angiogenesis and collagen structure.

In conclusion, our results suggest that, in contrast to liver fibrosis, necro-inflammatory activity may be better assessed with the multifrequency dispersion coefficient than with the monofrequency storage modulus. Hence three-dimensional multifrequency MR elastography, with its ability to generate both multi- and monofrequency parameters within a single examination could be a valuable tool for the non-invasive characterization of necro-inflammation and fibrosis in patients with chronic viral hepatitis.

## Methods

### Participants

Between November 2010 and June 2012, 50 consecutive patients with chronic viral hepatitis B or C scheduled for liver biopsy in the department of hepatology of our tertiary university hospital were prospectively included. The protocol was approved by the local institutional review board ("Comité d'évaluation de l'éthique des projets de recherche biomédicale (CEERB) Paris Nord", IRB 00006477) of the Hôpitaux Universitaires Paris Nord Val De Seine perimeter of Assistance Publique—Hôpitaux de Paris, and written informed consents were obtained. All work presented herein was performed in accordance with relevant guidelines/regulations and the Declaration of Helsinki. In this work, we report previously unexploited data about multifrequency MR elastography dispersion of the shear modulus. These data were acquired in a subcohort of patients (examined between November 2010 and June 2012) extracted from a larger cohort of patients (examined between November 2010 and October 2012). The patients in the larger cohort were examined with conventional monofrequency MR elastography and MR diffusion imaging. The results in these patients have been published^12^. The subcohort presented here was additionally imaged with the proposed multifrequency MR elastography sequence.

### MR elastography acquisition

MR elastography was performed in fasting patients with a 1.5 T MRI system (Intera, Philips Healthcare, Best, The Netherlands). T2-weighted MRI of the liver was performed for anatomical referencing. A gradient echo MR elastography sequence with fractional encoding was used (9 transverse slices with 4 mm thickness, 4–5 mm in-plane resolution depending on patient size, 9.6 ms echo time, 112 ms repetition time, 25° flip angle, 8 phase offsets, 3 encoded directions and a reference with mechanical vibration but no motion encoding)^16^. Synchronized mechanical vibrations of 28 Hz, 56 Hz and 84 Hz superimposed in one mechanical excitation were generated with an electromagnetic transducer (Philips Healthcare, Hamburg, Germany) placed against the right hypochondrium. The acquisition included four 19 s breath holds (supplementary material).

### MR data analysis

For MR elastography reconstruction, the shear, storage and loss moduli, as well as the damping ratio^38^ were calculated as described in the supplementary material. Only the 56 Hz frequency was considered for the single frequency analysis, as this frequency is closest to the reported frequency of 60 Hz often used in liver MR elastography^4^. The dimensionless multifrequency dispersion coefficient, γ, was calculated as described in the supplementary material.

The biomechanical parameters were measured by two physicists, PG and GP, with 8-year and 7-year expertise in abdominal MRI. The two physicists, who were blinded to the reference analyses, independently placed large regions of interest (ROIs) in the right liver, close to the transducer while avoiding large vessels and organ edges^39^ on three consecutive MR elastography magnitude images. The ROI size was 34.8 ± 17.6 cm^3^ and their location included the region of liver biopsy (segment 8). Datasets with < 3 µm of curl-filtered shear wave amplitude at any datasets were discarded.

### Histopathological and biological analyses

Liver biopsies were performed within two months of the MR elastography examinations. Hepatic necro-inflammation and fibrosis were assessed on the histological samples according to the METAVIR classification with necro-inflammatory activity graded as A0 = no activity, A1 = mild activity, A2 = moderate activity, and A3 = severe activity, and with fibrosis staged as F0 = no fibrosis, F1 = portal fibrosis without septa, F2 = portal fibrosis with some septa, F3 = fibrosis with numerous septa, and F4 = cirrhosis^40^. The biopsies were taken in hepatic segment 8.

The histological analysis was performed by a medical doctor with 25-year expertise in gastrointestinal histopathology. All patients had at least a liver specimen with more than 10 portal tracts. The pathologist was blinded to the clinical, MR elastography and biochemistry results. Serum alanine aminotransferase (ALT) and aspartate aminotransferase (AST) levels were measured at 37 °C within one week of the MR elastography acquisition.

### Statistical analysis

The sample size was calculated to allow at least 90% power to detect at a 5% significance level, a significant difference between the area under the receiver operating characteristic curve (AUC) of the multifrequency dispersion coefficient and a null hypothesis value of 0.50, considering the AUC of the dispersion coefficient = 0.80 for A ≥ 2, and the ratio of patients with A < 2/A ≥ 2 = 1.5, similar to the ratio reported by Poynard et al.^41^. Under these conditions, a sample size of 35 patients was required.

The inter-rater reproducibility was analysed with intraclass correlation coefficients and Bland–Altman bias and reproducibility indexes as previously defined ($1.96 \cdot \sqrt {2 \times percentage\; standard\; deviation}$^42^ Further analysis was carried out on the MR elastography measurements performed by the first reader.

Considering the small number of subjects in each METAVIR subgroup, the subjects were grouped in two activity classes, i.e. patients without (A0/A1) and with (A2/A3) substantial activity. For fibrosis severity, the participants were grouped in three classes with no/mild fibrosis (F0/F1), moderate fibrosis (F2), and severe fibrosis (F3/F4). Mann–Whitney and Kruskal–Wallis tests were respectively used to assess the differences in mechanical parameters between the inflammation and fibrosis classes.

The associations between necro-inflammatory activity, fibrosis, aminotransferase levels and viscoelastic parameters were assessed with Spearman rank correlation coefficients. Multivariate analysis was carried out with stepwise least squares multiple regression to investigate the influence of inflammation, fibrosis and aminotransferase levels on each biomechanical parameter. Parameters yielding *p* values greater than 0.1 were not retained. The partial regression coefficients and their associated *p* values were reported for the variables which were retained in the model.

The diagnostic performance of the viscoelastic parameters and aminotransferases serum levels was assessed with areas under the receiver operating characteristic curves (AUCs) and compared with the DeLong test. High diagnostic accuracy was considered for AUC > 0.8^43^. Sensitivity, specificity, positive and negative predictive values were calculated for the best viscoelastic parameters (storage modulus and multifrequency dispersion coefficient) using thresholds determined with Youden indexes to distinguish between different histopathological scores.

The results are expressed as mean ± standard deviation or median and 95% confidence interval. Significance was considered for *p* ≤ 0.05. The analyses were performed with Medcalc version 18.11.6_64 (Medcalc Software, Ostend, Belgium).

## Supplementary Information

Supplementary Information.

## Data Availability

The datasets generated during and/or analyzed during the current study are available from the corresponding author on reasonable request.

## Abbreviations

ALT: Alanine aminotransferase
AST: Aspartate aminotransferase
AUC: Area under the receiver operating characteristic curve
MR: Magnetic resonance
NASH: Nonalcoholic steatohepatitis
ROI: Region of interest
G′: Storage modulus
G″: Loss modulus
|G*|: Shear modulus
ζ: Damping ratio
γ: Multifrequency dispersion coefficient
