# Supplementary material for: Necro-inflammatory activity grading in chronic viral hepatitis with three-dimensional multifrequency MR elastography
Source: Sci Rep. 2021 Sep 29;11:19386. doi: 10.1038/s41598-021-98726-x (PMC8481240; doi:10.1038/s41598-021-98726-x)
Supplement: Supplementary file 1 — Supplementary Information. [file 41598_2021_98726_MOESM1_ESM.docx]

# SUPPLEMENTARY MATERIAL

# Supplementary tables

Supplementary table 1: Distribution of fibrosis stages and necro-inflammatory activity grades according to the METAVIR scoring system

| \|  \| A0 \| A1 \| A2 \| A3 \| **Total** \| \| --- \| --- \| --- \| --- \| --- \| --- \| \| F0 \| 4 \| 1 \| 0 \| 0 \| **5** \| \| F1 \| 3 \| 14 \| 2 \| 0 \| **19** \| \| F2 \| 0 \| 4 \| 3 \| 2 \| **9** \| \| F3 \| 0 \| 5 \| 3 \| 1 \| **9** \| \| F4 \| 0 \| 3 \| 2 \| 0 \| **5** \| \| **Total** \| **7** \| **27** \| **10** \| **3** \| **47** \| |  |
| --- | --- | --- | --- | --- | --- | --- | --- | --- | --- | --- | --- | --- | --- | --- | --- | --- | --- | --- | --- | --- | --- | --- | --- | --- | --- | --- | --- | --- | --- | --- | --- | --- | --- | --- | --- | --- | --- | --- | --- | --- | --- | --- | --- |

Data are number of patients

Supplementary table 2: Characteristics of the biomechanical parameters and serum aminotransferase levels in necro-inflammatory activity grading and fibrosis staging

|  | A ≥ 1 | | | A ≥ 2 | | | A = 3 | | |
| --- | --- | --- | --- | --- | --- | --- | --- | --- | --- |
|  | Se / Sp | PPV / NPV | threshold | Se / Sp | PPV / NPV | threshold | Se / Sp | PPV / NPV | threshold |
| G' | 100 / 73 | 20 / 100 | 2.35 kPa | 62 / 79 | 53 / 84 | 2.35 kPa | 100 / 73 | 20 / 100 | 2.35 kPa |
| G'' | 55 / 71 | 92 / 22 | 1.12 kPa | 54 / 65 | 37 / 79 | 1.16 kPa | 67 / 89 | 29 / 98 | 1.88 kPa |
| G* | 38 / 100 | 100 / 22 | 2.87 kPa | 69 / 62 | 41 / 84 | 2.63 kPa | 100 / 57 | 14 / 100 | 2.64 kPa |
| ζ | 60 / 71 | 92 / 24 | 0.27 | 77 / 53 | 39 / 86 | 0.27 | 67 / 80 | 18 / 97 | 0.24 |
| γ | 90 / 71 | 95 / 56 | 1.40 | 100 / 68 | 54 / 100 | 1.21 | 100 / 84 | 30 / 100 | 1.00 |
| ALT | 83 / 86 | 97 / 46 | 54.5 U/L | 54 / 88 | 64 / 83 | 148 U / L | 100 / 57 | 14 / 100 | 82 U / L |
| AST | 70 / 86 | 97 / 33 | 40 U/L | 77 / 79 | 59 / 90 | 60 U / L | 100 / 68 | 18 / 100 | 60 U / L |
|  |  |  |  |  |  |  |  |  |  |
|  |  |  |  |  |  |  |  |  |  |
|  | F ≥ 2 | | | F ≥ 3 | | | F = 4 | | |
|  | Se / Sp | PPV / NPV | threshold | Se / Sp | PPV / NPV | threshold | Se / Sp | PPV / NPV | threshold |
| G' | 83 / 96 | 95 / 85 | 2.25 kPa | 64 / 94 | 82 / 86 | 2.66 kPa | 100 / 95 | 71 / 100 | 3.42 kPa |
| G'' | 74 / 79 | 76 / 73 | 1.13 kPa | 86 / 70 | 55 / 92 | 1.13 kPa | 100 / 83 | 42 / 100 | 1.44 kPa |
| \|G*\| | 87 / 83 | 83 / 87 | 2.62 kPa | 64 / 88 | 69 / 85 | 2.89 kPa | 100 / 95 | 71 / 100 | 3.98 kPa |
| ζ | 65 / 58 | 60 / 64 | 0.27 | 79 / 48 | 39 / 84 | 0.27 | 80 / 52 | 17 / 96 | 0.26 |
| γ | 83 / 71 | 73 / 81 | 1.24 | 93 / 61 | 50 / 95 | 1.24 | 80 / 52 | 17 / 96 | 1.21 |
| ALT | 78 / 83 | 82 / 80 | 82 U / L | 86 / 73 | 57 / 92 | 83 U / L | 80 / 76 | 29 / 97 | 115 U / L |
| AST | 70 / 92 | 89 / 76 | 55 U / L | 79 / 85 | 69 / 90 | 63 U / L | 80 / 81 | 33 / 97 | 76 U / L |

Note. G’: storage modulus, G’’: loss modulus, |G*|: shear modulus, ζ: damping ratio, γ: multifrequency dispersion coefficient, Se: sensitivity, Sp: specificity, PPV: positive predictive value; NPV: negative predictive value.

# Supplementary figures

## Figure S1:


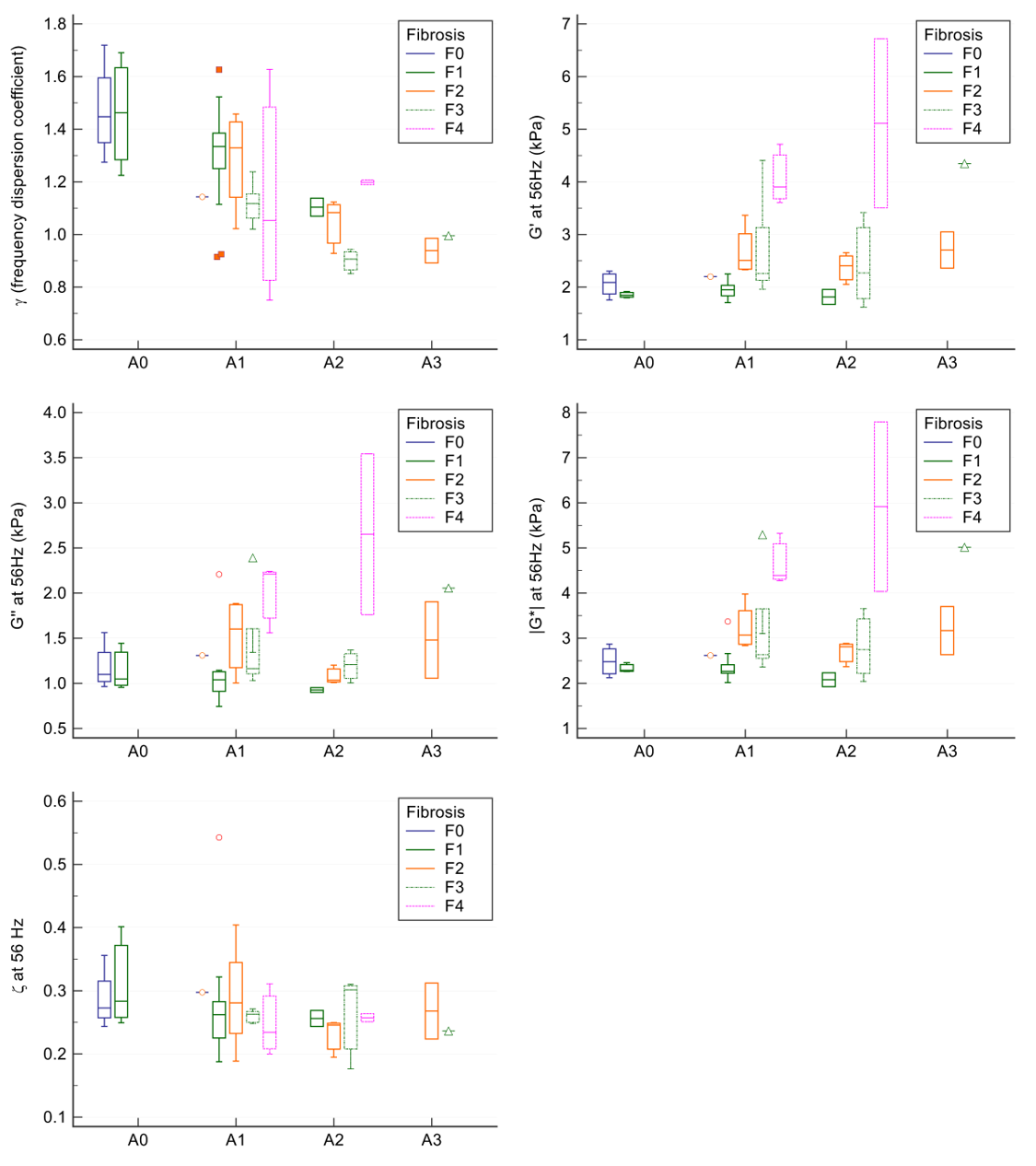


Boxplots (boxes: 1st to 3rd quartiles, whiskers: 1st quartile - 1.5 × interquartile range to 3rd quartile + 1.5 × interquartile range, horizontal line: median) of multifrequency dispersion coefficient (γ, panel A), storage modulus (G’, panel B), loss modulus (G’’, panel C), shear modulus (IG*I, panel D) and damping ratio (ζ, panel E) at 56Hz in patients with increasing necro-inflammation score, subdivided relative to fibrosis scores.

## Figure S2:


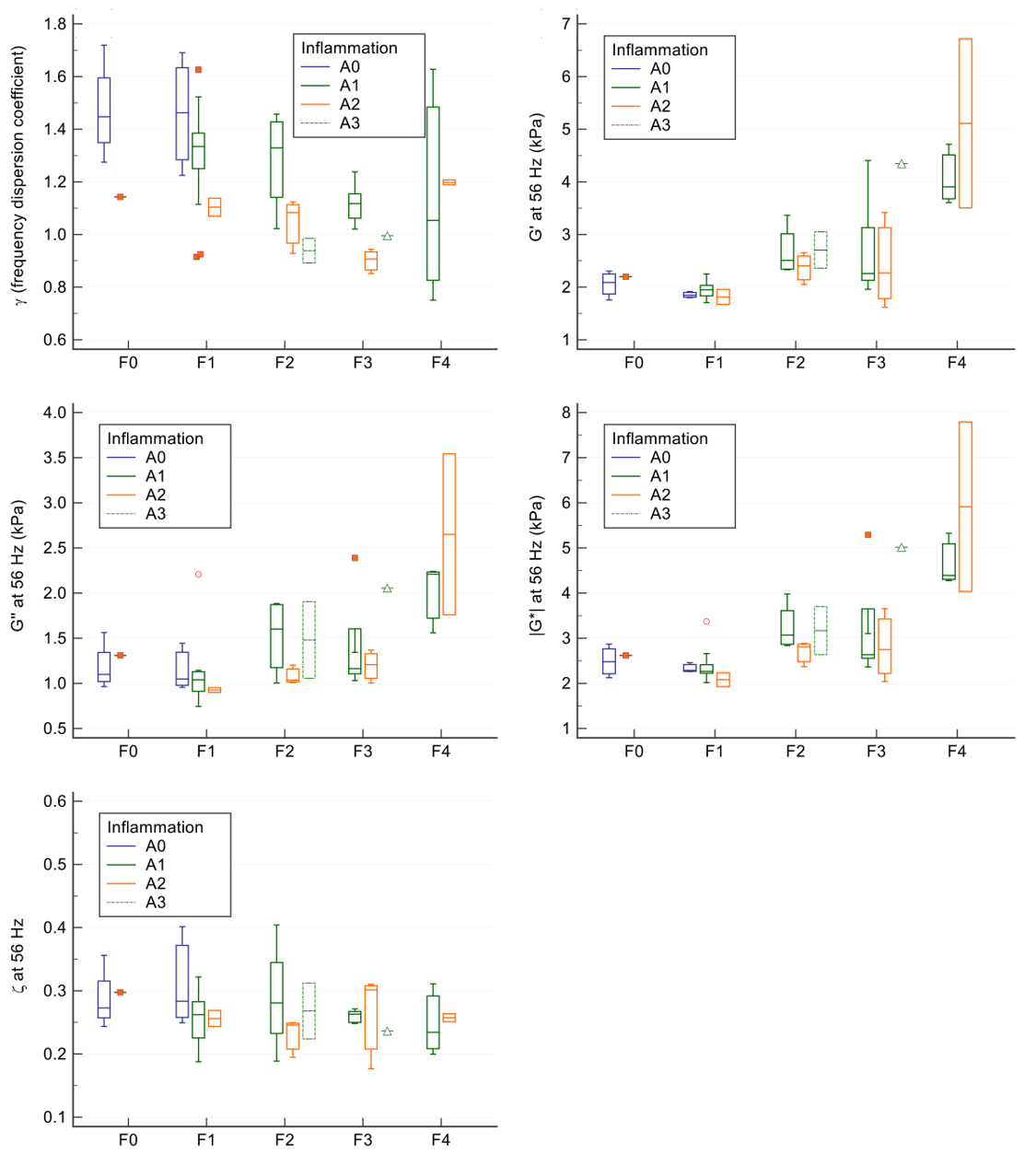


Boxplots (boxes: 1^st^ to 3^rd^ quartiles, whiskers: 1^st^ quartile - 1.5 × interquartile range to 3^rd^ quartile + 1.5 × interquartile range, horizontal line: median) of multifrequency dispersion coefficient (γ, panel A) and monofrequency (56 Hz) storage modulus (G’, panel B), loss modulus (G’’, panel C), shear modulus (IG*I, panel D) and damping ratio (ζ, panel E) in patients with increasing fibrosis. For each fibrosis group, boxplots are subdivided relative to the necro-inflammatory activity.

# Supplementary methods

## MR elastography Acquisition

Motion-sensitization gradients were added to a gradient echo sequence. Echo time was set at the second in-phase condition for water and fat spin populations (TE = 9.6 ms), thereby making it unnecessary to adopt fat suppression schemes. This duration, considering the duration required for slice selection, phase encoding and the required gradient slew times, enabled a period of 8.3 ms for motion sensitization. Hence, encoding was performed with motion-sensitization gradients at 120 Hz to maximize the encoding efficiency while keeping a TE of 9.6 ms, thereby conserving acceptable levels of signal to noise ratio considering the short T2* time of liver tissue.

Mechanical vibration was generated with an electromagnetic-mechanical transducer positioned on the right hypochondrium of the patient. The transducer was phase-locked to the MR system, and emitted a waveform comprising a superposition of equally weighted sinusoidal signals with no phase offsets at 28, 56 and 84 Hz throughout the acquisition. Encoding was performed in a fractional mode (34), with encoding ratios of 0.70, 0.47 and 0.23, respectively, corresponding to encoding efficiencies of 149 µm/rad, 82 µm/rad and 66 µm/rad, respectively. Magnitude and phase MR images of the instantaneous wave pattern were obtained at eight mechanical phase offsets across the period of the lowest frequency component of the waveform. These eight multi-slice images were acquired sequentially for three mutually orthogonal directions of phase encoding. A reference scan with no motion encoding was also acquired.

## Calculation of the visco-elastic parameters

The complex viscoelastic parameters were calculated by first separating the frequency components of the wave field with discrete time Fourier transform. The, inversion of the linear viscoelastic complex three dimensional wave equation (25, 35) was carried out separately at each frequency. The biomechanical properties that were computed were the shear modulus magnitude IG*I, the storage modulus (real part of G*: G'), the loss modulus (imaginary part of G*: G'') and the damping ratio (ζ, calculated with ζ = G’’/2G’) at 56 Hz. For the multifrequency analysis, parametric maps of |G*| were calculated separately at each frequency. Model independent frequency dispersion of the shear modulus was assessed by assuming a power law (i.e. $|G^{*}|\left( \omega\right)\propto A\cdot{}^{Ὑ}$) with |G*|(ω) being the frequency-dependent shear modulus, γ (gamma) the power law exponent or frequency dispersion coefficient, ω the angular frequency and A a scaling parameter (16). The exponent of the frequency term was calculated by analytical regression (linear regression in log-log space) using as datapoints the ROI averages calculated at each frequency.
